# Elevated serum CA 19–9 level mimicking pancreaticobiliary carcinoma from a hepatic abscess: case report and literature review

**DOI:** 10.3389/fmed.2024.1470046

**Published:** 2025-01-14

**Authors:** Shaurya Dhingra, Puneet Raman, Taylor Ramsaroop, Isaiah Harrison, Tova Bergsten, Erin Nusbaum, Lawrence E. Feldman

**Affiliations:** ^1^University of Illinois College of Medicine, Chicago, IL, United States; ^2^Jesse Brown VA Medical Center, Chicago, IL, United States

**Keywords:** CA 19–9, tumor marker, hepatic abscess, hepatobiliary, hepatology

## Abstract

Serum levels of the tumor marker CA 19–9 are widely utilized in the diagnosis and monitoring pancreatic and biliary malignancies. However, serum levels of CA 19–9 have also been reportedly elevated in non-malignant conditions. Here, we present the rare case of a 65-year-old woman with a history of gallbladder malignancy who was found to have a new hepatic lesion on surveillance CT with an associated elevation in CA 19–9 to 5,866 U/mL. Drainage of the lesion and treatment with antibiotics resulted in a rapid decline in CA 19–9 levels, indicating that the elevation in CA 19–9 was due to a benign hepatic lesion. We review eight similar reported cases of CA 19–9 elevations due to benign hepatic abscesses, thereby highlighting a need to interpret the tumor marker with caution.

## Introduction

CA 19–9 is normally produced in both biliary and pancreatic ductal cells, as well as epithelial cells of gastric, colonic, endometrial, and salivary origin (1). As a serum tumor marker for pancreatic carcinoma, CA 19–9 levels are ascertained at the time of diagnosis and for subsequent monitoring of disease activity. The potential efficacy of this marker in the diagnosis of pancreatic cancer has been extensively studied (2, 3). Furthermore, the sensitivity and specificity in symptomatic patients with pancreatic cancer has been reported to range from 70–79% and 68–91%, respectively (4, 5). This marker is also commonly employed in the management of biliary tract cancers such as cholangiocarcinoma and gallbladder cancer. For example, the sensitivity and specificity of an elevated serum CA 19–9 level in the diagnosis of cholangiocarcinoma has been reported to be similar to that of pancreatic cancer (6). Outside of pancreaticobiliary malignancies, serum CA 19–9 has been reported to have varying levels of utility and remains frequently used in the management of other gastrointestinal malignancies including cancers of the esophagus, stomach, gall bladder, and liver (7).

Increased levels of serum CA 19–9 have been observed in non-malignant conditions. Benign biliary conditions causing cholestasis or obstruction, such as acute cholangitis and choledocholithiasis, have been well documented to cause elevations in levels of serum CA 19–9 (7–9). Benign non-pancreaticobiliary conditions have been reported to cause CA 19–9 elevations. In one retrospective study of 192 individuals analyzing non-malignant and non-pancreaticobiliary causes of elevations in CA 19–9, diseases of the lungs, gynecological tract, and endocrine system were identified as among the many sources of an elevated serum CA 19–9 level (10). However, in this study, hepatic disease represented the largest percentage of cases, accounting for 32.8% of cases. Within this cohort, elevation of serum CA 19–9 levels were attributable to alcoholic cirrhosis, various forms of hepatitis, and in one instance, a hepatic cyst. In many cases, the serum CA 19–9 levels decreased or normalized in concert with improvement in liver function tests.

Hepatic abscess remains one of the least common diagnoses to be reported as associated with an increase in the serum CA 19–9 level. In our literature review, we found only eight known case reports detailing this association. Herein, we report a case of a patient with history of gallbladder cancer presenting with a rising serum CA19-9 level due to a hepatic abscess mimicking a recurrence of her cancer. We also review the literature for any prior case reports of hepatic abscess as an etiology for an elevated serum CA19-9 level.

### Case report

A 65-year-old woman with a history of mixed T2N0M0 (stage IIB) adenocarcinoma and T3N1M0 (stage IIIB) neuroendocrine carcinoma of the gallbladder presented to the oncology clinic for follow up. Nine years prior, her cancer had been discovered incidentally during a cholecystectomy performed for symptomatic cholelithiasis. She subsequently underwent a secondary radical procedure consisting of a regional lymphadenectomy, hepaticojejunostomy, Roux-en-Y, and resection of the liver, gallbladder bed, and bile duct, including stump of the cystic duct followed by adjuvant chemotherapy and radiation therapy. CT scans after completion of adjuvant therapy demonstrated no evidence of disease. Moreover, surgical pathology showed no evidence of malignancy in specimens taken from the radical procedure.

**Figure 1:**
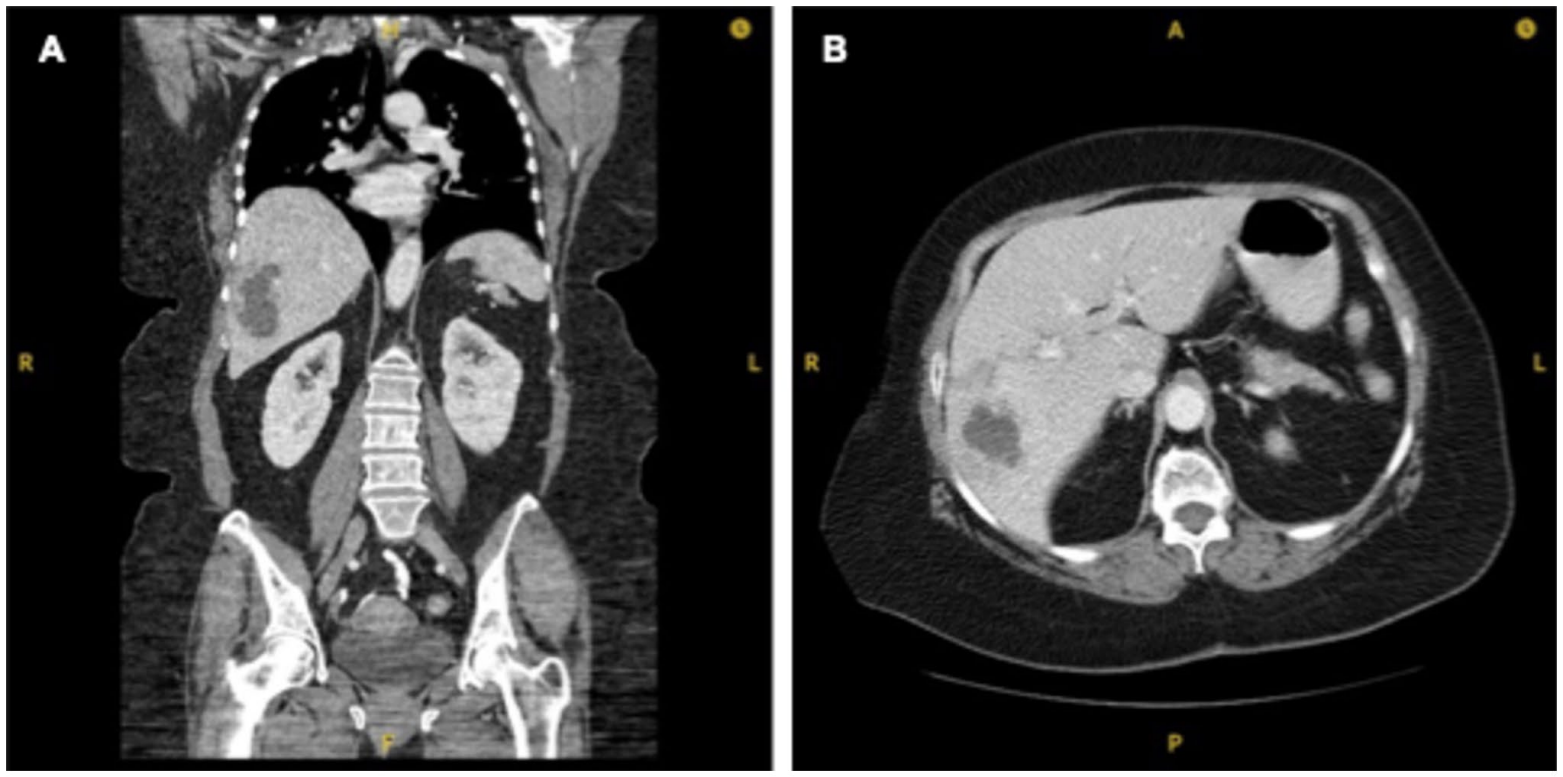
CT chest abdomen and pelvis with 4.4 × 7.1 × 6.0 cm multi-cystic lesion in right hepatic lobe. **(A)** Coronal view. **(B)** Axial view.

Upon current presentation for annual surveillance, CT scan demonstrated a finding of a 4.4 × 7.1 × 6.0 cm multi-cystic lesion in the right hepatic lobe (Figure 1). The patient denied any constitutional symptoms, including subjective fevers and abdominal pain. She remained afebrile, and routine labs, including hepatic function tests, were unremarkable except for moderate thrombocytosis. Notably, however, 1 week prior to this scan, the patient had presented to an outside hospital with complaints of fevers, chills, night sweats, and right-upper quadrant abdominal pain. Labs had shown a mild leukocytosis, but liver function tests were normal. She was empirically treated for suspected gastroenteritis with a five-day course of amoxicillin and discharged. Tumor marker labs obtained at the time of surveillance imaging resulted to show an isolated increased serum CA 19–9 level of 5,866 U/mL, elevated from 15 U/mL 1 year prior. Other serum tumor markers including carcinoembryonic antigen (CEA), chromogranin A, and Alpha Fetoprotein (AFP) were within normal limits. The patient was subsequently admitted for elevated CA 19–9 in the setting of a hepatic mass, with a differential diagnosis that included malignancy and hepatic abscess.

Ultrasound guided aspiration of the intrahepatic lesion yielded frankly purulent fluid, favoring diagnosis of hepatic abscess and requiring the placement of a percutaneous drainage catheter. One day after drainage, repeat CA 19–9 was found to have decreased to 2,398 U/mL. Aspirate cytology revealed predominantly inflammatory cells and culture of the material returned positive for *Citrobacter braakii*. The patient was started on intravenous piperacillin and tazobactam at first, but after return of susceptibilities, switched to 2-week course of oral levofloxacin and metronidazole. The intrahepatic abscess drain output remained between 10 and 50 mL per day for the remainder of hospitalization, while serum CA 19–9 levels trended down to 1,281 U/mL, 784 U/mL, and finally 435 U/mL at three, four, and 5 days after drain placement, respectively. On the fifth day, the patient was discharged home with the drain in place.

Repeat CT 8 days after discharge showed a hypoattenuating area decreased in size from previous scans. Sonographic and fluoroscopic examinations during the drain removal procedure confirmed resolution of the abscess. On clinical follow up 2 weeks later, the serum CA 19–9 level elevation had completely resolved, with the tumor marker measuring at a level of only 34 U/mL. The patient reported no recurrence of symptoms in the interim period. Another month later, magnetic resonance cholangiopancreatography confirmed near complete resolution of the abscess.

### Review of case reports

We performed a literature search of case reports in which CA 19–9 was elevated due to a hepatic abscess and found a total of eight reported cases (Table 1). The mean peak CA 19–9 in these cases was 1,538 (range 115–6,000). Three of these cases presented with fever and CT scans revealed hepatic lesions that were determined to be abscesses growing *E coli*. One was a 59-year-old man that was undergoing adjuvant treatment for pancreatic ductal adenocarcinoma. The second case was a 66-year-old woman with a history of hepatic hydatid cyst resection. The third case was that of an 87-year-old woman with no reported medical history (11–13). In all three cases, antibiotic treatment resulted in symptomatic improvement and a subsequent decline in CA 19–9 levels.

**Table 1:** Previously reported cases of elevated CA 19–9 due to hepatic abscess without malignancy.

Year	Authors	Age	Sex	Organism	Peak serum CA 19–9 (U/mL)
2005	Soardo et al. (17)	31	M	*Actinomyces*	278
2006	Giannaris et al. (12)	66	F	*E. coli*	6,000
2012	Yoshino et al. (11)	87	F	*E. coli*	851
2014	Sohaib et al. (14)	85	F	MRSA and *Klebsiella*	1,387
2015	Lui et al. (15)	71	M	Gram positive cocci in chains	115
2019	Denis et al. (16)	35	M	Unknown	3,399
2019	Marin-Leiva et al. (18)	53	M	*Entamoeba Histolytica*	117
2021	Arivazhagan et al. (13)	59	M	*E. coli*	158

Normal range: 0–37 U/mL.

Organisms other than *E. coli* causing hepatic abscesses with an associated elevation in CA 19–9 have also been reported. An 85-year-old woman with a history of breast cancer had *Klebsiella* and MRSA cultured from a complex cystic mass in the liver as seen on ultrasound and magnetic resonance imaging (MRI) (14). A 71-year-old man with a history of a cholecystectomy had Gram-positive cocci in chains observed in drainage from a multiloculated liver abscess, though cultures were ultimately negative (15). Both individuals initially presented with fevers and elevated CA 19–9 levels and had symptomatic improvement after treatment with antibiotic therapy. A decline in CA 19–9 levels was reported for the 85-year-old woman, but no details were provided in the case of the 71-year-old man. In a separate case report, a 35-year-old male with cystic fibrosis and a history of lung, liver, and renal transplantation was hospitalized with a fever (16). After a negative infectious workup, an elevated CA 19–9 level was observed, and MRI findings showed multiple hepatic lesions resembling liver abscesses. Culture findings were not reported, but CA 19–9 declined, and symptoms resolved after antibiotic treatment.

Surgical excision of hepatic segments containing abscesses have also reportedly resulted in resolution of symptoms. A 31-year-old male presented with fevers, and ultrasound and CT scans revealed the presence of multiple hepatic lesions. *Actinomyces* spp. were found to be responsible for multiple liver abscesses and an associated increase in CA 19–9 (17). The diagnosis of *Actinomyces* was confirmed after surgical resection of hepatic segments IV-VII from which yellow pus was obtained and cultured. Symptoms improved rapidly after resection and antibiotics, and CA 19–9 levels normalized.

Only a single case of non-bacterial liver abscess causing elevated CA 19–9 has been reported. A 53-year-old male from Peru presented with fever, abdominal pain, nausea, and jaundice (18). A CT scan revealed a hepatic mass, and CA 19–9 was elevated. The patient developed septic shock and underwent exploratory laparotomy showing an abscessed hepatic mass. Hepatic biopsy performed during the procedure showed amoebic trophozoites. Following surgical and antibiotic treatment, the patient’s clinical status improved. CA 19–9 levels after treatment were not reported.

## Discussion

This case report adds to the rare instances in which the CA 19–9 tumor marker has been reported to be elevated due to a hepatic abscess. The tumor marker is routinely used as a diagnostic tool for pancreatic and biliary malignancies, and to evaluate the malignancy’s response to the treatment. The CA 19–9 level reported in this case was significantly elevated, nearing the high end of the range of all previously reported cases. The markedly high levels of the tumor marker strongly suggested either recurrence of gall bladder cancer, or a new second primary hepatobiliary cancer. The rapid decline in the serum CA 19–9 levels after drainage was key in signifying an abscess as the etiology of the elevated CA 19–9 level, as opposed to recurrence of malignancy.

The process by which benign conditions cause elevations in CA 19–9 remains unclear. However, it is believed that the presence of biliary stasis or inflammation can lead to obstruction of the biliary ducts, which in turn induces production of the tumor marker (9). In this case, as in the other cases reviewed here, obstruction of biliary ducts and associated biliary stasis induced by a hepatic abscess may have been responsible for elevated levels of CA 19–9. After successful treatment of the abscess, the levels of CA 19–9 in the serum returned to their normal range, pointing toward the resolution of the hepatobiliary obstruction. As is the case in malignancy, serum CA 19–9 levels in this instance also served as an indicator to assess the effectiveness of the treatment, with levels dropping rapidly just 1 day after abscess drainage.

Our study reiterates that tumor markers such as CA 19–9 must be interpreted carefully and with appropriate clinical context. Benign conditions, like the one described in this case, can cause serum CA 19–9 levels to rise to heights similar to those seen in malignant cases, making it difficult to use the marker to distinguish between benign and malignant conditions. Further study is required into the mechanism by which such abscesses induce elevations in CA 19–9 levels.

## Data Availability

The original contributions presented in the study are included in the article/supplementary material, further inquiries can be directed to the corresponding author.
